# Molecular and Functional Characterization of *FLOWERING LOCUS T* Homologs in *Allium cepa*

**DOI:** 10.3390/molecules21020217

**Published:** 2016-02-16

**Authors:** Ranjith Kumar Manoharan, Jeong Suk Hyeon Han, Harshavardhanan Vijayakumar, Boopathi Subramani, Senthil Kumar Thamilarasan, Jong-In Park, Ill-Sup Nou

**Affiliations:** 1 Department of Horticulture, Sunchon National University, 255, Jungang-ro, Suncheon, Jeonam-do 57922, Korea; mrkumarbiotech@gmail.com (R.K.M.); han-402@hanmail.net (J.S.H.H.); vharshavardhanan@gmail.com (H.V.); senkuttybio@gmail.com (S.K.T.); jipark@sunchon.ac.kr (J.-I.P.); 2 Research Center for Applied Sciences, Academia Sinica, Nangang, Taipei 11529, Taiwan; boopathi@sinica.edu.tw

**Keywords:** *Allium cepa*, *FLOWERING LOCUS T*, modelling, gene expression, contrasting line

## Abstract

Onion bulbing is an important agricultural trait affecting economic value and is regulated by flowering-related genes. *FLOWERING LOCUS T (FT)*-like gene function is crucial for the initiation of flowering in various plant species and also in asexual reproduction in tuber plants. By employing various computational analysis using RNA-Seq data, we identified eight *FT*-like genes (*AcFT*) encoding PEBP (phosphatidylethanolamine-binding protein) domains in *Allium cepa*. Sequence and phylogenetic analyses of FT-like proteins revealed six proteins that were identical to previously reported AcFT1-6 proteins, as well as one (AcFT7) with a highly conserved region shared with AcFT6 and another (comp106231) with low similarity to MFT protein, but containing a PEBP domain. Homology modelling of AcFT7 proteins showed similar structures and conservation of amino acids crucial for function in AtFT (*Arabidopsis*) and Hd3a (rice), with variation in the C-terminal region. Further, we analyzed *AcFT* expression patterns in different transitional stages, as well as under SD (short-day), LD (long-day), and drought treatment in two contrasting genotypic lines EM (early maturation, 36101) and LM (late maturation, 36122). The *FT* transcript levels were greatly affected by various environmental factors such as photoperiod, temperature and drought. Our results suggest that *AcFT7* is a member of the *FT*-like genes in *Allium cepa* and may be involved in regulation of onion bulbing, similar to other *FT* genes. In addition, *AcFT4* and *AcFT7* could be involved in establishing the difference in timing of bulb maturity between the two contrasting onion lines.

## 1. Introduction

Angiosperms, a diverse group of flowering plants, have evolved to carry out complex developmental processes, from vegetative to reproductive growth, that are greatly affected by endogenous and environmental factors [1,2]. The flowering cycle is regulated by various factors and pathways including photoperiod, vernalization, gibberellins, ambient temperature, age and autonomous pathways [3,4]. Flowering occurs following a period of vernalization [5] and is influenced by three major genes, *VRN1*, *VRN2*, and *FLOWERING LOCUS T* (*FT*, also known as *VRN3*) [6]. The *FT* gene was first identified in *Arabidopsis thaliana* [7,8], in which it plays a major role in the vernalization and photoperiod pathway and in initiating the flowering signal in the apical meristem. Previous study in model plant *Arabidopsis* revealed translocation of FT proteins, a component of florigen [9], to the apical meristem from leaf tissues. Furthermore, FT proteins induce flowering after long distance transport, thus acting as an mobile flowering signal [10]. Additionally, CONSTANS (CO) acts as transcriptional activator by binding to DNA via conserved TGTG(N2-3)ATG motif. Gene organization of *FT* genes shown that affinity of *CO* genes to bind and regulating *FT* genes is predominant due to tandem duplication of their conserved motifs in their promoter region [11], thus act as photoperiod regulators of *FT* genes [12]. *FT* genes encode proteins with a phosphatidylethanolamine-binding protein (PEBP; [13,14]) domain that interacts with the bZIP transcription factor *FLOWERING LOCUS D* (FD) at the shoot apical meristem (SAM) and initiates flowering [15,16]. This FT-FD protein complex activates other flower development-related genes such as *APETALA1* and *LEAFY* [17,18]. Studies in *Arabidopsis* have elucidated that *FT* is a member of a small gene family in the group of six members: *TERMINAL FLOWER1* (*TFL1*), *TWIN SISTER OF FT* (*TSF*), *BROTHER OF FT AND TFL1* (*BFT*), *Arabidopsis thaliana CENTRORADIALIS HOMOLOGUE* (*ATC*), and *MOTHER OF FT AND TFL1* (*MFT*). However, the 3D structures of FT and TFL1 proteins reveal that they have antagonistic functions during flowering period [19,20]. Recent studies in other species revealed that some FT homologs induce flowering such as in rice [21], wheat [22], barley [23,24], sugarcane [25], lettuce [26], potato [27], jatropha [28], pineapple [29], spring orchid [30], sugar beet [31], longan [32], onion [33], tomato [34], maize [35], *etc.*

*Allium cepa* (onion), a (2n = 16) dicot member of the Asparagales, is one of the most widely consumed vegetables, and can show great variation in characteristics such as size, color, shape, and pungency. During onion growth, the leaf scale thickens to form the characteristic bulb, which represents an overwintering vegetative stage in their life cycle. In addition, *FT* genes are involved in a range of physiological processes, including bulb development in onion [33] and tuberization in potato [36]. For bulb initiation, onion leaves must be exposed to inductive photoperiod conditions. It was first reported by Garner and Allard that bulbs develop in response to long day (LD) photoperiods. However, in tropical regions, short day (SD) onions develop bulbs in response to short day photoperiods. Like flowering, bulb formation and growth are greatly influenced by photoperiod and temperature [37]. Furthermore, monocots such as in rice and maize, have almost three to four times more PEBP-encoding genes than *Arabidopsis* [21,24,35]. This complexity in the PEBP gene family in monocots suggests that the functions of this family in monocots are complicated than in the dicot *Arabidopsis thaliana*. To date, a total of six *A. cepa* genes (*AcFT1-6*) have been identified and characterized with regard to bulb formation and vernalization. Among the six *FT* genes, *AcFT2* regulates flowering and *AcFT4* suppress bulb formation whereas *AcFT1* promotes bulbing [33]. 

In this study, we performed computational analysis employing gene prediction tools (Hidden Markov model, HMM) to identify novel genes using RNA-seq data in *Allium cepa*. Further we analyzed *FT* genes to clarify their functional roles in onion. We also set out to investigate and compare expression patterns of PEBP domain-containing FT proteins and monitor their expression profiles during various growth stages in two contrasting lines, an early-maturation line (EM, 36101) and late-maturation line (LM, 36122). We report the expression profiles of *FT* genes under LD and SD photoperiods and during drought stress. Our aim was to identify novel *FT* genes in *A. cepa* and to find potential genes underlying the differences in maturation cycle between the two contrasting lines.

## 2. Results and Discussion

### 2.1. Search for Onion FT Genes and Phylogenetic Analyses

In both plant and animal kingdoms, researchers use well-established gene prediction tools based on specific protein domain searches to identify candidate genes from large datasets. Currently, a completely sequenced onion genome is not available and studies related to the identification of new PEBP family members in *A. cepa* are limited. Hence, we set out to investigate new PEBP family proteins among annotated proteins from two *A. cepa* lines 36101 and 36122, and performed BLAST searches using HMM profiles of PEBP domains as queries. As onion has a limited number of transcript sequences available so far, we also performed an *in-silico* search to identify *FT* genes from the RNA-Seq data of transcripts assembled from the leaf tissues of the two Korean onion lines 36101 and 36122 (SRP064878). To extend our search for new *FT* genes, we used a gene prediction tool (HMMER) to identify candidate *FT* genes based on their protein domain (PEBP domain) structure. In total, we used 89 aligned orthologous FT proteins (Table S1) from different species as an input for gene prediction in the *A. cepa* RNA dataset. We obtained 12 redundant proteins that contained the PEBP domain in their protein structure. Finally, we obtained eight non-redundant proteins after removing duplicates. Redundancy in our results shows that more than one copy of *FT* genes encoding PEBP domains exists in the transcriptome data. Out of the eight non-redundant proteins, six corresponded to the FT-like (AcFT1 to AcFT6) proteins identified by Lee *et al.* [33]. The other two represented novel PEBP-encoding genes (comp32886 and comp106231) identified with HMM-based prediction tool. A homology search for orthologous genes using the BLASTP program confirmed that these two proteins belong to PEBP containing protein family, and that their functional annotations were similar. In addition, sequence similarity of the eight proteins was analyzed using pairwise alignment performed with DiAlign [38]. The two proteins showed less similarity to the other FT-like proteins. However, based on annotation from RNA-Seq data, comp32886 showed the highest similarity (66%) to FT6 protein and comp106231 showed 79% similarity to an uncharacterized protein from *Elaeis guineensis* (Table S3). Domain analysis results from CDD and SMART databases confirmed the presence of the PEBP domain in the N-terminal regions of these proteins, and the secondary structure was predicted by the EMBOSS program [39]; details are provided in Table S4. The sizes of the corresponding predicted proteins ranged between 175 and 202 amino acids. The ranges for protein length, molecular weight, pI and instability index were 18.36–23.05 kDa, 5.20–8.79 and 29.97–51.23, respectively. AcFT6 and comp106231 were predicted to be stable and all other proteins were predicted to be unstable. Detailed secondary structures are predicted and tabulated in Table S4.

**Figure 1:**
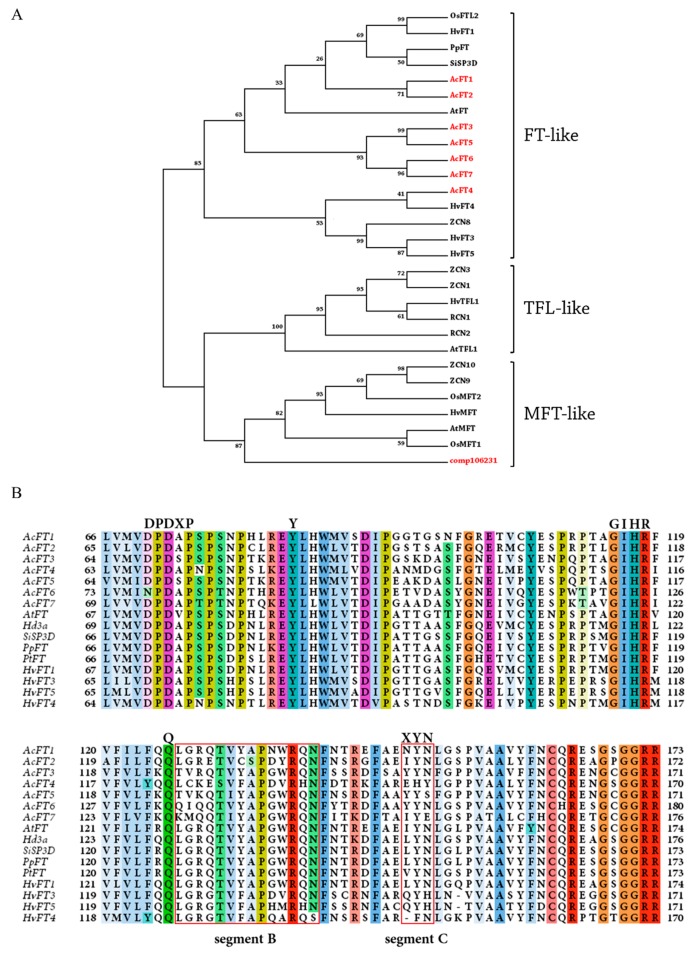
(**A**) Phylogenetic analysis of members of PEBP family from rice, barley, peach, maize, and *Arabidopsis*. Sequences were aligned using ClustalW, and tree was constructed using Maximum likelihood (ML) method employing JTT matrix along with 1000 bootstrap replication. The bootstrap value at the tree nodes represent robustness of the tree. Red color highlighted genes represent identified FT proteins in *Allium cepa*; (**B**) Alignment of the deduced protein sequence of AcFT, identical residues are shaded with different colors. Solid red boxes represent Segment B (14-amino acid length) and C (XYN triad), respectively, and capitalized letters above the alignment represent functionally important conserved residues of FT proteins.

Previous phylogenetic analysis has led to classification of PEBP-containing plant proteins into three main clades: FT-like (induce flowering), TFL1-like (suppress flowering) and MFT-like (induce flowering) [40]. In tomato and maize, apart from their role in flowering, PEBP-domain proteins act as general growth regulators [34,41], as illustrated by Pnueli *et al.* [42] for tomato SELF PRUNING (SP), a TFL1 homolog that can interact with variety of proteins. Phylogenetic analysis of deduced amino acid sequences of the *FT-like*, *TFL* and *MFT* genes in *A. cepa*, *Arabidopsis*, barley, rice, peach, tomato and maize (Table S5) revealed that comp32886 belongs to the FT-like clade as expected; however comp106231 belongs to the clade MFT (Figure 1A). Despite the presence of a PEBP domain in their protein structure this latter protein shares very low similarity and few conserved residues with other MFT proteins (e.g., barley MFT-22%), so we omitted comp106231 from further analysis. The annotation for comp106231 based on BLAST results was as an uncharacterized protein, and the putative function of this PEBP domain-containing protein remains unknown. We named the other novel protein (comp32886) as *AcFT7*. The deduced amino acid and coding DNA sequence of AcFT7 predicted using computational tools are listed in Table S6.

FT protein forms a complex (Florigen activation complex) with 14-3-3 proteins and bZIP binding protein transcription factor FD. Differences in FT protein activities might be due to their binding affinity towards 14-3-3 proteins. Protein sequence alignment to known FT-like homologs from other plant species revealed that AcFT7 has a conserved histidine residue at position 88 (H88) (Figure 1B), similar to other AcFT proteins reported by Lee *et al.* [33]. In *Arabidopsis*, this aromatic histidine residue is at the key position determining whether the protein is a floral promoter (FT-like) or repressor (TFL1-like) [43]. Generally, FT proteins contain a specific region called segment B (14 amino-acid length), which forms an additional loop in the protein structure, and is necessary for FT proteins to function as floral promoters [19]. Specifically, the key residues Gln140, Asp144, Glu141 in segment B in FT, TFL1 and BFT, respectively, determine FT-like or TFL1-like activity [19,20].

Additionally, FT and TFL1 proteins contain a triad region called segment C (LYN triad) [19]. FT proteins require both segment B and segment C for full functionality, whereas TFL1-like activity remains unaltered if either segment is mutated or deleted. However, variation in segment C might modify the functionality of the protein, such as from promoter to repressor or *vice versa*. In *A. cepa* cultivar CUDH2107, AcFT1 activity was suppressed by AcFT4 expression thus inhibits bulb formation [33]. This overpowering activity of AcFT4 might be due to variation within segments B (residues 145, 146 and 147) and C, since segments B and C are critical for FT-like activity.

**Figure 2:**
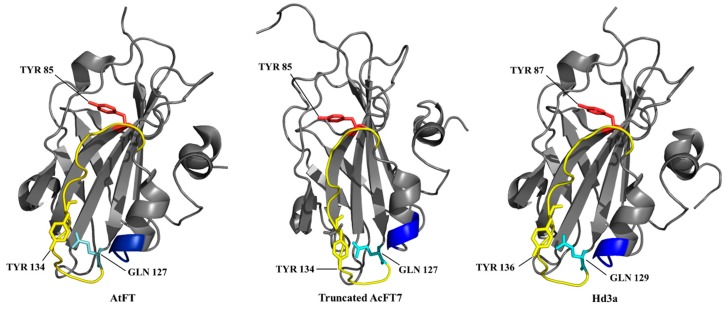
Predicted homology protein model of truncated AcFT7 in comparison to AtFT (*Arabidopsis*; 1AWF) and Hd3a (rice; 3AXY_A) protein. The external loop segment B is represented in yellow and segment C (XYN triad) in blue color. The crucial residues Tyr (red), Gln (cyan), Tyr (yellow in Segment B) are shown as sticks.

Thus, AcFT4 and AcFT1 might be antagonistic to one another in *A. cepa*. Similar antagonistic FT-like homolog pair been demonstrated in sugar beet [31,44]. We constructed a truncated 3D model of AcFT7 protein in MODELLER based on the similarity with crystal structure of *FLOWERING LOCUS T* (FT) from *Arabidopsis thaliana* (1WKP; [19]) and *Oryza sativa* (3AXY_A; [45]). AcFT7 sequence shared 52% and 50% identity with 1WKP and 3AXY_A structures. The superimposition of AcFT7 protein with templates showed only variation at the C-terminal, with an extended loop (Figure 2). An anion-binding site was present in the central β-sheet in the structural model, and was found in other FT family proteins of plants [20,32,33,43,45,46]. Overall, *in-silico* analysis based on sequence information, evolutionary relationships and homology modeling of AcFT7 protein revealed that it has considerable similarity to orthologous FT proteins in plant species.

### 2.2. Gene Expression Correlates with Bulb Initiation

In onion, the physiology of bulb initiation was broadly described by Mettananda and Fordham [47]; photoperiodic conditions affect bulb initiation, similar to photoperiodic control of flowering in other species [48,49,50]. A recent study revealed that flowering genes of *Arabidopsis* involved in day length responses are functionally conserved with regard to those involved in onion flowering and bulbing [51]. Research in various species has revealed that *FT*-like proteins act during developmental processes such as termination of meristem growth and tomato yield [52,53], tuberization in potato [36], termination of growth in poplar trees [54], plant architecture in maize [41], stomatal control [55] and reproductive architecture in *Arabidopsis* [25]. Hence, it was considered likely that the genes controlling photoperiodic flowering also control bulbing in onion [33]. Bulb initiation and development vary between genotypes based on responses to different environmental conditions and on the length of time spent during various developmental stages in onion. To discover whether any of the *FT* genes are potentially involved in regulating bulb formation, two contrasting inbred lines of onion were used in this work, we examined the expression of *FT* genes during three stages of plant development: seedling stage (SS), bulb formation (BF), and bulb maturity (BM). Characterization of the seven *FT* genes were carried out using expression analysis in two different onion lines 36101 (EM, early maturation) and 36122 (LM, late maturation) under greenhouse conditions. Here we provide evidence that *FT* genes play a crucial role in bulb development in two contrasting inbred lines that are phenotypical similar, but have genetic variations.

**Figure 3:**
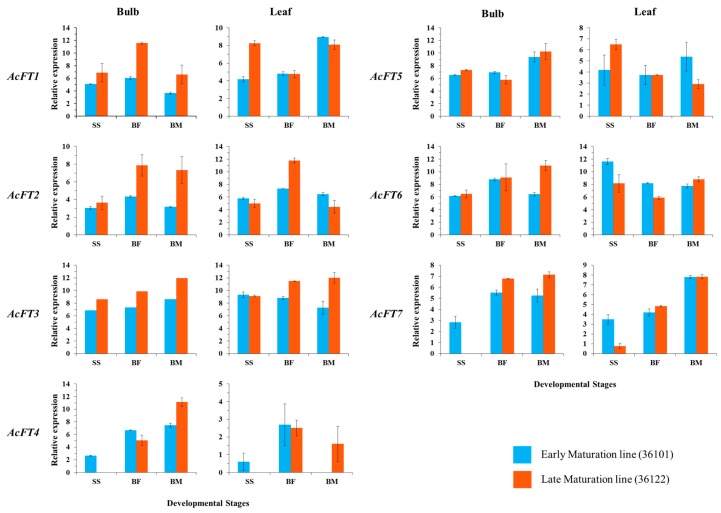
Expression of *AcFT* genes in EM (early maturation, 36101) and LM line (late maturation, 36122), leaf and bulb tissues were harvested during three stages: seedling stage (SS), Bulb formation (BF) and Bulb maturation (BM). Data represent an average ± s.e.m of three biological replicates, with transcripts normalized to *β-tubulin*.

To investigate the expression of *FT* genes, bulb and leaf tissues were harvested at three stages: seedling stage (SS), bulb formation (BF), bulb maturity (BM) in two contrasting onion inbred lines grown in greenhouse conditions. All *FT* genes were ubiquitously expressed in all stages of investigated tissues (leaves and bulb) except *AcFT4* and *AcFT7* (Figure 3). *AcFT1* showed an exponential decrease in transcript levels between the transition phases (Seedling to bulbing) in leaf tissues but increased levels in bulb tissues. These findings suggest that *AcFT1* is predominantly involved in bulbing, similar to previous findings from onion grown in SD and LD photoperiods [33]. Unlike *AcFT1*, strong induction of *AcFT2* transcript levels occurred during bulb formation in bulb and leaf tissues of the two lines, whereas it was downregulated during bulb maturation stage in bulb tissues. In onion, flowering genes are highly expressed in emerging floral bud tissues after vernalization [33]. Consistent with this, its expression pattern in bulb tissues of the two lines suggests that *AcFT2* acts as a floral regulator despite of the genetic variation during flowering period of these two lines. Furthermore, in both tissues the expression of *AcFT4* and *AcFT7* was either not detected or at very low levels at the seedling stage in the LM line; however in EM line both genes showed significant expression. Additionally, expression of these *AcFT4* and *AcFT7* genes in bulb tissues drastically increased when the plants entered the bulbing period, but *AcFT7* showed static expression during bulb maturity period. 

These expression patterns suggest that *AcFT4* and *AcFT7* could possibly be involved in bulb development under greenhouse conditions. These two genes might be involved in bulb formation in the studied short-day inbred onion lines which requires short day length to promote bulbing. However in CUDH2107 onion cultivar, *AcFT4* gene expression is down-regulated along with increased expression of *AcFT1* when the onion plants were shifted from short day to long day photoperiod [33]. Bulb initiation could be altered by the lack of expression of *AcFT4* and *AcFT7* in the LM line, whereas their expression might underlie the earlier bulbing in the EM line. Additionally, in our experimental study genotype and environmental factors may influence the expression of FT genes and also bulb formation. Among the *FT* genes, *AcFT3*, *AcFT5* and *AcFT6* exhibited similar expression patterns with no significant difference in their transcript levels during plant development. Nevertheless, these genes might still have different functions as differences in FT protein functionality in plant tissues are largely affected by their interactions with 14-3-3 protein, which together with FD transcription factor forms the complex florigen to initiate flowering [45]. Also, flowering and bulbing in onion are influenced by temperature and photoperiodic conditions [37,56,57,58], which affect the expression of *FT* genes under greenhouse conditions. Overall, these results indicate that *AcFT4* and *AcFT7* could possibly be the major genes involved in bulb formation and in the late maturity of the LM line under greenhouse conditions.

### 2.3. Photoperiod Control in Bulb Formation

Environmental conditions such as temperature and day length influence bulb formation and flavour quality in onion [59,60]. In onion, light spectrum quality influences bulb formation [56,61]. Other *Allium* species including garlic, also have been reported to be influenced by temperature, day length and carbohydrate contents in terms of bulb induction and development [62,63,64]. Previous researchers reported that plants exposed to shorter day lengths than they require will form only leaves without bulbs [65] and in some cases thick bulb necks may also occur [58]. Conversely, premature bulb formation, bulb development, and maturity rates increase when plants are exposed to longer day lengths than they require; this leads to small bulbs and low yield [57].

**Figure 4:**
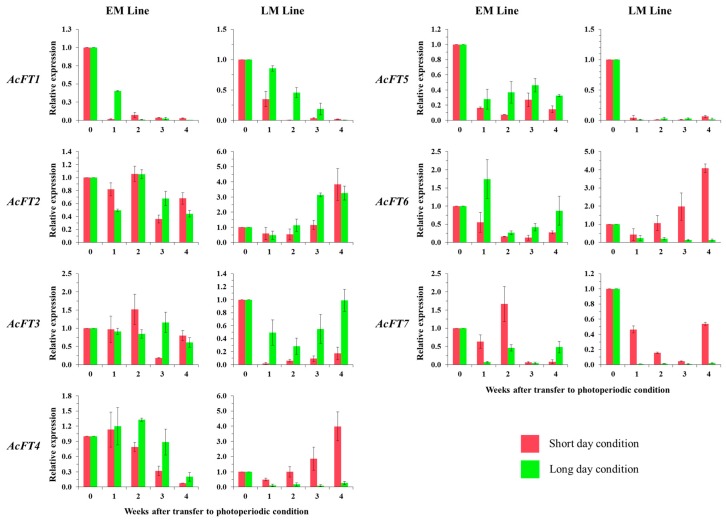
Expression of *AcFT* genes in EM (early maturation, 36101) and LM line (late maturation, 36122), bulb tissues were studied during Bulb maturation stages under SD (short day) and LD (long day) photoperiodic condition. In the graph, x axis represent samples at various durations of photoperiod treatment (0 to 4 weeks), y axis represent relative expression of *FT* genes. Data represent an average ± s.e.m of three biological replicates, with transcripts normalized to *β-tubulin.*

Based on these considerations, we set out to investigate whether the genes we identified could be involved in bulb initiation during different photoperiod conditions (SD and LD) or/and greenhouse conditions. Onion lines were exposed to two different photoperiod conditions and the expression patterns in bulb samples were observed after bulb initiation. Overall, *FT* genes from EM line were similar under both SD and LD conditions and did not appear to be differentially expressed during differing photoperiod conditions except *AcFT1* and *AcFT4* (Figure 4). Moreover, *AcFT2* showed differences in expression pattern in both lines under the different photoperiod conditions compared to the normal greenhouse conditions. This result indicates that *AcFT2* is strongly affected by day-length conditions, but the expression patterns confirmed the differences in bulb formation under different environmental conditions [37]. The mRNA levels of *AcFT1* genes of both lines were downregulated when the plants were placed under both photoperiod conditions. Further, *AcFT1* and *AcFT4* were down-regulated in EM line under both SD and LD conditions towards bulb maturity and this was in contrast to the greenhouse conditions. However, *AcFT4* was upregulated during SD conditions in the LM line; this could be due to genetic differences related to bulbing time in the two onion lines. These results thus provide further evidence that *AcFT4* could be involved in bulb formation and suggest that *AcFT4* activity might depend on internal factors.

The functionality of the *AcFT* genes could be altered by external factors such as light intensity and temperature within the growth room [66]. Interestingly, some genes were strongly affected by the day-length conditions in the LM line, and we observed that expression fluctuated weekly in a photoperiod-dependent manner. *AcFT4* and *AcFT6* genes were upregulated towards bulb maturation in SD, but showed very low expression in the LD conditions. Our evidence indicates that in greenhouse and under SD conditions (8h light: 16h dark), *AcFT4* functions likely remain the same in the EM line (Figure 4). By contrast, *AcFT7* was influenced by photoperiod in both lines. This suggests that when LM line reaches critical LD length *AcFT4* and *AcFT1* transcript levels are decreased and increased respectively, when compared to SD condition, thus promoting bulb formation [33]. Further work will be needed to understand the initiation and inhibitory activity of *AcFT4* during SD and LD conditions, in bulbing and flowering, respectively. We propose that increased expression of *AcFT4* in SD conditions may also be involved in the late maturity of the LM line.

Okporie and Ekpe [67] reported that bulbing occurred largely under LD conditions in onion. A similar pattern related to flowering has been observed in other plants, especially *Arabidopsis*, in which *FT* was upregulated when plants were exposed to LD photoperiod conditions [10]. Tuberization in potato is controlled by the photoperiod response to SD [68], suggesting that the genetic control is similar in tuberization and photoperiodic flowering. Previous findings clearly show that *FT* genes are related to flowering [10]. *FT* genes are conserved in species including rice [21], tomato [34], Darnel ryegrass [69], sugar beet [31], and wheat [70]. Our results further show that expression of *FT* genes controls bulbing and also plant maturity in onion lines.

### 2.4. FT Gene Expression in Drought Stress

**Figure 5:**
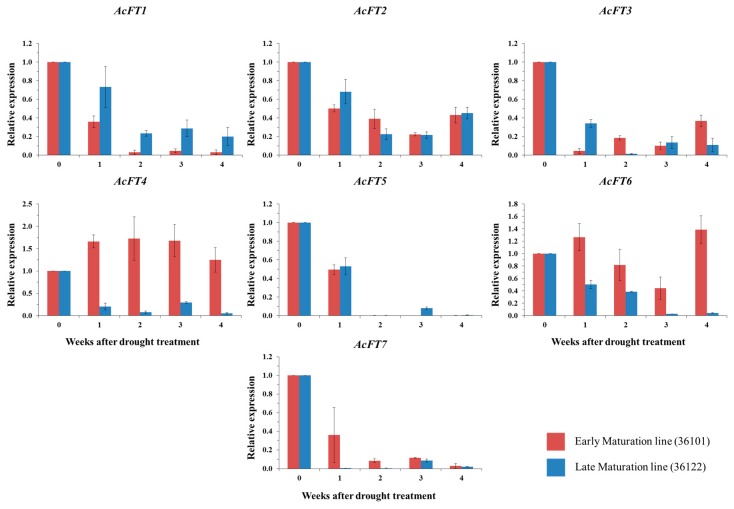
Expression of *AcFT* genes in EM (early maturation, 36101) and LM line (late maturation, 36122), bulb tissues were studied during Bulb maturation stages under drought condition. In the graph, x axis represent samples at various durations of drought stress (0 to 4 weeks), y axis represent relative expression of *FT* genes. Data represent an average ± s.e.m of three biological replicates, with transcripts normalized to *β-tubulin*.

Abiotic stresses such as drought, salt and temperature stress can affect plant growth and development and cause severe damage to cell membranes, resulting in reduction of yield [71]. Recent studies indicated that drought stress is involved in the upregulation of the florigen genes during bud development and flowering in the tropical tree *Shorea beccariana* [72]. *FT* genes are upregulated by drought in *Arabidopsis* after 4 to 5 days of treatment [73,74]. However, it is unclear how drought affects gene expression during bulb formation in *A. cepa*. To determine whether *FT* genes were responsive to drought, we examined the expression of the seven *FT* genes in the two onion lines after the bulb initiation stage. We found that all *FT* genes responded to drought during bulb formation in both lines (Figure 5). Notably, among all the *FT* genes in the EM line, only *AcFT4* expression under drought conditions increased, although it was downregulated in the LM line. Similar *FT* gene expression during drought conditions was observed related to flowering in the non-model plant *S. beccariana* [72]. *AcFT6* in the EM line showed downregulation and a drastic change in expression at the end of the treatment, whereas the remaining *FT* genes were downregulated in both the EM and LM lines during the entire treatment. Significantly, *AcFT5* and *AcFT7* were strongly inhibited during drought stress. Our findings are consistent with reports that *FT* genes are responsive to drought and epigenetic changes in *Arabidopsis* [74,75]. Taken together with previous reports, these results suggest complex effects on *FT* genes during drought that function during floral organ development and bulb initiation. In summary, onion plant adaptation to drought stress is complex, and this study could help efforts to improve crop yield under drought stress.

## 3. Materials and Methods

### 3.1. Plant Material

For the experimental study, two contrasting short-day onion inbred lines 36101 (EM, early maturation) and 36122 (LM, late maturation) were purchased from the Nongwoo Seed Company, Suwon, Korea. The seeds were sown in early autumn season (September 2014). Sixty-two plants with 3–4 leaves (65 days old seedlings) of each line were transplanted during late autumn season (November 2014) and grown until harvesting stage in greenhouse (November to June—9 to 13 h day length period) at Sunchon National University. Plants were transferred to growth room to study the effects of photoperiod condition on *FT* genes when they reached bulb formation stage, 180 and 210 days old plants for EM and LM, respectively. These plants were grown for four weeks under either long-day (LD) or short-day (SD) photoperiod conditions of 16 h light and 8 h light, respectively in a plant growth room maintained at a light intensity of ≈115 µmol·m^−2^s^−1^ and 20 °C with 30%–40% humidity. Simultaneously, twelve plants of the same age from each line were grown under drought conditions for four weeks in the greenhouse under intermediate day length (11–13 h light), whereas control plants received regular watering. Three biological replicates were used for each analysis in this study.

### 3.2. Sequence Analysis

To identify *FT* genes from our RNA-Seq data (Sequence Retrieval Archive accession number: SRP064878) *Allium cepa*, we carried out computational analysis using HMMER 3.1b2 software [76] with FT-specific domain (PEBP domain) sequences of orthologous FT proteins. We used orthologous FT proteins containing the PEBP domain region as a training set for HMMBUILD and HMMSEARCH program. Resultant protein sequences were analyzed for the presence of typical PEBP domains using the CDD database [77,78] and SMART database [79,80]. Phylogenetic analyses were performed using deduced amino acid sequences, and alignment of the PEBP domains from orthologous PEBP genes in barley, rice, *Arabidopsis*, peach, tomato and maize was conducted using ClustalX [81,82] The evolutionary tree was constructed using the Maximum-Likelihood (ML) method with the JTT (Jones, Taylor, Thornton; [83]) matrix via MEGA6.06 software [84]. Bootstrap analysis for 1000 replicates was performed to provide confidence estimates for the tree topologies and relatedness. We performed primary analysis of the predicted molecular weights, pIs, and stability indexes using ProtParam [85]. Secondary structures of FT proteins were predicted using the garnier script tool from EMBOSS-6.6.0 [86]. The homology model was built by using MODELLER integrated with UCSF Chimera (ver. 1.10.2) [87] using potential templates (1WKP, *Arabidopsis thaliana*; 3AXY_A, *Oryza sativa*) [19,45] from the PDB database.

### 3.3. Analysis of Gene Expression

Three different stages; seedling stage (SS, 65 days old, 3–4 leaves), bulb formation (BF, 180 and 210 days old plants for EM and LM line respectively) and bulb maturation (BM, 215 and 280 days old plants for EM and LM line respectively) were analyzed for stage-specific expression analysis of *FT* genes. Leaf and bulb tissues (SS—root collar tissues, BF—premature bulb tissues, BM—mature bulb tissues) were harvested during respectively stages. Bulb tissues during bulb formation stage (BF) was collected from SD-, LD- and drought-treated samples during different time courses (0–4 weeks) and stored using liquid nitrogen. Total RNA was extracted using the RNeasy Mini Kit (Qiagen, Valencia, CA, USA) according to the manufacturer’s instructions. RNA concentrations were determined using a ND 1000 (Nano Drop, Wilmington, DE, USA). The extracted RNA was treated with RNase-free DNase to remove genomic DNA contaminants (Qiagen). cDNA was subsequently synthesized using a Superscript^®^ III First-Strand Synthesis Kit (Invitrogen, Carlsbad, CA, USA) according to the manufacturer’s instructions. Real-time PCR was performed using the Roche LC96 machine and Light-cycler 96 SYBR Green I Master mix (Roche, Basel, Switzerland). Relative gene expression levels were calculated using the ∆∆CT method. *β-tubulin* was used as housekeeping gene. Primer pairs used for quantitative reverse transcriptase-PCR experiments can be found in Table S2.

## 4. Conclusions

In conclusion, we identified a novel *FT* gene and reported its structural variation compared to other *FT* genes. In addition, we carried out expression analysis in two bulb onion lines with different genotypic origins, implying the presence of differences in these lines specific for maturation time. Our data suggest that *FT* genes could play roles in bulb formation in both lines and in addition to *AcFT1* reported by Lee *et al.* [33] *AcFT4* and *AcFT7* might be also involved in early and late bulb initiation in the onion lines studied.

## Supplementary Materials

Supplementary materials can be accessed at: http://www.mdpi.com/1420-3049/21/2/217/s1.
